# Hyperandrogenism in a Postmenopausal Woman Secondary to Testosterone Secreting Ovarian Stromal Tumor with Acoustic Schwannoma

**DOI:** 10.1155/2018/8154513

**Published:** 2018-12-05

**Authors:** Bindu Gandrapu, Preeyanka Sundar, Brian Phillips

**Affiliations:** ^1^Department of Internal Medicine, Berkshire Medical Center, Pittsfield, MA, USA; ^2^Division Director, Department of Endocrinology, Berkshire Medical Center, Pittsfield, MA, USA

## Abstract

Androgen-secreting ovarian neoplasms are rare ovarian tumors that present with hirsutism and virilization which may manifest as severe alopecia, deepening of voice, and clitoromegaly. Most often, ovarian tumors are found to be very small or even undetectable. In such cases, bilateral salpingo-oophorectomy should be performed after ruling out other causes of high androgens. We present a 63-year-old postmenopausal woman with clinically and radiologically undetectable testosterone-secreting ovarian tumor, which was later on detected on biopsy.

## 1. Introduction

Hyperandrogenism refers to the condition in which excessive androgens are found circulating in the female body. Clinical manifestations of hyperandrogenism include hirsutism, alopecia, acne, virilization, and emotional distress [1]. Common causes of hyperandrogenism are polycystic ovary syndrome (PCOS), congenital adrenal hyperplasia (CAH), Cushing's syndrome, and ovarian tumors [2]. At reproductive age, PCOS is considered the most frequent cause of high androgens in females [2]. Virilization significantly affects the quality of life, posing a management challenge [1]. In postmenopausal woman, the patients with hirsutism and signs of virilization are often associated with ovarian or adrenal tumors [3]. Androgen secreting neoplasms are rare ovarian tumors that account of 5% of all ovarian tumors [4]. Common androgen secreting ovarian tumors include Leydig cell neoplasm, stromal luteoma, Sertoli-Leydig cell tumor, and ovarian hyperthecosis [5]. Most often, these tumours present with virilization, raised testosterone level, and bilateral salpingo-oophorectomy being indicated after ruling out adrenal neoplasm [1]. We present a postmenopausal woman with testosterone secreting ovarian stromal tumor that was clinically and radiologically undetectable and was only confirmed by biopsy after bilateral salpingo-oophorectomy.

## 2. Case Presentation

**Figure 1:**
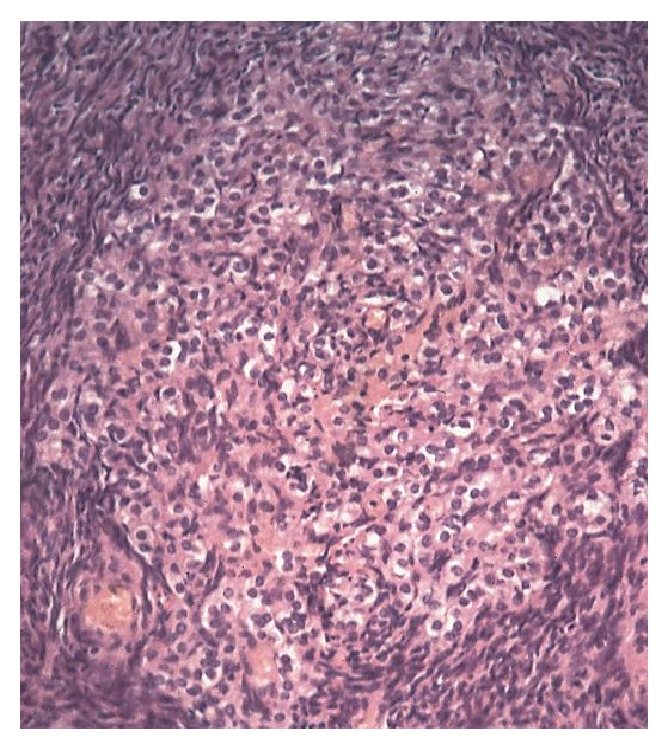
Large nodule of luteinized cells is present within the ovarian stroma (nodular hyperthecosis).

A 63-year-old postmenopausal woman presented with deepening of voice, and increased hair growth on her face and lower abdomen over the past few months. She noticed thinning of her hair a few years ago. She was sexually active up until last year. She complained of decreased libido, disturbed sleep, back pain, right ear deafness and urge incontinence for years. She had a 36-year-old son and a 33-year-old daughter. She developed menopause on early 50s. Past history included hypertension, obstructive sleep apnea, tonsillectomy, and tubal ligation. She had family history of chronic kidney disease, hypertension, malignant neoplasm of urinary bladder, malignant melanoma of skin, myelodysplastic syndrome, and sudden death. On clinical examination, blood pressure was 132/76 mmHg and heart rate was 64/m. She was anxious and overweight (BMI: 38.06) with enlarged thyroid gland, clitoromegaly, male pattern baldness (significant loss of scalp hair) and hirsutism. Laboratory reports showed normal urea (27 mg/dL) and creatinine (1.45 mg/dL), elevated testosterone (210 ng/dL; normal: 12-72 ng/dL), raised DHEA-S (235 *μ*g/dL), hyperlipidemia, normal TSH (1.09 IU/mL), LH, FSH and estradiol. Abdominal ultrasound scan and uterine echotexture were normal and Pap smear was negative. CT scan brain showed normal pituitary gland. MRI brain and internal auditory canal showed a 2.1 x 1.1 x 1 x 1 cm right acoustic schwannoma in the internal auditory canal with extension into the cerebellopontine angle cistern with involvement of the right cochlea and the vestibule with no evidence of pituitary tumor or brain compression. Elevated testosterone settled after the trial of Leuprolide. Diagnosis of hyperandrogenism was made and bilateral salpingo-oophorectomy was performed. Bilateral laparoscopic salpingo-oophorectomy revealed left stromal luteoma, bilateral stromal nodular hyperthecosis (see Figure 1), and right paratubular cysts. However, uterine cavity was normal in size, nontender and mobile. MRI adrenals without contrast were normal. Testosterone secreting ovarian tumour was suspected.

## 3. Discussion

Androgen-secreting ovarian tumors are a rare cause of elevated testosterone in postmenopausal women, accounting for 5% of all ovarian neoplasms. Most often, these tumors are too small to be detected clinically or imaging radiology as seen in our case. In our case, ultrasound scan, CT scan, and MRI imaging gave no clue to elevated testosterone. It is important to distinguish the source of androgens in order to improve the classification, the understanding of androgen excess disorders, and subsequent management [6]. Then as a trial, Lupron may cause decline in the level of testosterone, giving an impression of gonadotropin-responsive testosterone-secreting ovarian tumor. Lupron is a GnRH agonist, which on constant presence by depo causes ovarian depression after initial brisk stimulation [7]. Later on, bilateral laparoscopic salpingo-oophorectomy and excisional biopsy revealed left stromal luteoma, bilateral stromal hyperthecosis and right paratubular cysts. Various case studies are available where ultrasound scan, CT scan, MRI imaging failed to detect androgen-producing ovarian tumors [8, 9]. Therefore, detection, localization and removal of androgen-secreting tumors are of significant importance.

Ovarian hyperthecosis is a rare nonmalignant entity encountered in postmenopausal hyperandrogenism [1]. Other ovarian tumors include steroid cell tumor, ovarian luteoma, and Leydig cell tumor. Hyperthecosis is a condition where ovarian stroma possesses nests of luteinized theca cells that produce large amounts of androgens [10]. The characteristics of hyperthecosis include severe hyperandrogenism, insulin resistance, hirsutism, and virilization [11]. Ovarian hyperthecosis is also associated with hyperestrogenism, hyperinsulinemia, and hyperlipidemia [1]. In our case, elevated testosterone, hirsutism, virilization, prediabetes and hyperlipidemia favour ovarian hyperthecosis, which was confirmed on biopsy. Dolinko and Ginsburg presented a similar case of a 69-year-old woman where they failed to locate the elevated androgen source and the patient was treated with leuprolide [9]. They did not perform oophorectomy due to the poor condition of the patient. Similarly, postmenopausal androgen-producing stromal luteoma of ovary is also a rare tumor. Bogdanou et al. [11] reported similar case of ovarian luteoma. Lee et al. [12] reported a 70-year-old woman with multiple comorbidities who presented with progressive hirsutism and bilateral temporal balding. Tumor survey only detected elevated testosterone and renal vein catheterization identified right ovarian androgen-secreting tumor. The patient was not fit for any surgery and so was put on gonadotropin-releasing hormone-agonist (GnRH-a) for 6 months which resulted in drop in testosterone level to normal limit. Hence, GnRH-a can be used immediately if surgical procedures are unsuitable for the patient. Similarly, Wang et al. [13] suggested GnRH-a as an alternative for the management of steroid cell tumor of ovary.

The present study reports a rare androgen-secreting ovarian hyperthecosis and stromal luteoma, which was not detected, on USG, CT scan, and MRI imaging. Failure of imaging techniques led to the trial of leuprolide and bilateral laparoscopic salpingo-oophorectomy. This case study demonstrates the importance of investigation of elevated testosterone in a postmenopausal woman when there is no evidence of adrenal tumor.

## 4. Conclusion

We describe a postmenopausal woman with an androgen secreting ovarian tumors with elevated testosterone levels which may be undetectable on clinical and radiological examination. In such cases, the patient should be thoroughly investigated ruling out all other causes of elevated androgens before undergoing bilateral salpingo-oophorectomy.
